# Low Dose Pig Anti-Influenza Virus Monoclonal Antibodies Reduce Lung Pathology but Do Not Prevent Virus Shedding

**DOI:** 10.3389/fimmu.2021.790918

**Published:** 2021-12-16

**Authors:** Basudev Paudyal, Adam McNee, Pramila Rijal, B. Veronica Carr, Alejandro Nunez, John McCauley, Rodney S. Daniels, Alain R. Townsend, John A. Hammond, Elma Tchilian

**Affiliations:** ^1^ Host Responses, The Pirbright Institute, Pirbright, United Kingdom; ^2^ Centre for Translational Immunology, Chinese Academy of Medical Sciences Oxford Institute, University of Oxford, Oxford, United Kingdom; ^3^ Medical Research and Council (MRC) Human Immunology Unit, MRC Weatherall Institute of Molecular Medicine, Radcliffe Department of Medicine, University of Oxford, Oxford, United Kingdom; ^4^ Department of Pathology and Animal Sciences, Animal and Plant Health Agency-Weybridge, Addlestone, United Kingdom; ^5^ Worldwide Influenza Centre, The Francis Crick Institute, London, United Kingdom

**Keywords:** influenza monoclonal antibody, monoclonal antibody therapy, swine, mucosal immunity, pandemic H1N1 virus

## Abstract

We have established the pig, a large natural host animal for influenza, with many physiological similarities to humans, as a robust model for testing the therapeutic potential of monoclonal antibodies (mAbs). In this study we demonstrated that prophylactic intravenous administration of 15 mg/kg of porcine mAb pb18, against the K160–163 site of the hemagglutinin, significantly reduced lung pathology and nasal virus shedding and eliminated virus from the lung of pigs following H1N1pdm09 challenge. When given at 1 mg/kg, pb18 significantly reduced lung pathology and lung and BAL virus loads, but not nasal shedding. Similarly, when pb18 was given in combination with pb27, which recognized the K130 site, at 1 mg/kg each, lung virus load and pathology were reduced, although without an apparent additive or synergistic effect. No evidence for mAb driven virus evolution was detected. These data indicate that intravenous administration of high doses was required to reduce nasal virus shedding, although this was inconsistent and seldom complete. In contrast, the effect on lung pathology and lung virus load is consistent and is also seen at a one log lower dose, strongly indicating that a lower dose might be sufficient to reduce severity of disease, but for prevention of transmission other measures would be needed.

## Introduction

Influenza virus infection remains a global health threat to humans and animal influenza A viruses are the CDC’s top zoonotic pathogen. Although influenza vaccines are available, most inactivated and live-attenuated influenza vaccines are strain specific, requiring constant updating, and influenza is still a cause of serious morbidity and mortality in high-risk groups, such as young children, the elderly and immunocompromised patients (1). Monoclonal antibodies (mAbs) have been proposed as a strategy to provide immediate immunity to combat virus infections including influenza and both prophylactic and therapeutic treatments of influenza with mAbs have been shown to be effective in mice and ferrets but results in the clinic have been mixed. While polyclonal antibody preparations have shown disease-modifying activity (2), results of clinical trials with broadly neutralizing (bn) anti-hemagglutinin (HA) stalk mAbs such as MEDI18852 and MHAA4549A have been disappointing (3–7). However, the lack of efficacy in clinical trials may be obscured by variability due to pre-existing immunity in human experimental challenge or natural infection studies and the difficulty of achieving a therapeutic effect in the face of a high viral load. Furthermore, most people with early infection recover and therefore it is difficult to demonstrate an effect compared to placebo. Likewise, it may be difficult to demonstrate a benefit in patients with a more severe disease, in whom inflammation and lung pathology, which are difficult to assess in humans, may be more important than viral replication. An additional problem is the difficulty in achieving a high concentration of mAb in serum and the respiratory tract in humans with a large body mass.

There is therefore a need for a large animal model in which mAbs were selected on the basis of *in vitro* studies or efficacy in small animals can be further tested. Pigs can provide such a model. Pig anatomy and physiology closely resembles that of humans. They have a similar distribution of sialic acid receptors in their respiratory tract and are infected with similar influenza A viruses, making them a powerful natural host large animal model to study immunity to influenza (8, 9). We have previously shown that the strongly neutralizing human IgG1 monoclonal antibody, 2-12C, administered prophylactically at 15 mg/kg to pigs, reduced virus load and lung pathology after H1N1pdm09 challenge (10). However, the study could not be extended beyond 5–6 days due to the development of anti-human IgG responses. To overcome this limitation, we have isolated porcine mAbs from H1N1pdm09 infected pigs (11). The porcine antibodies were directed towards the two major immunodominant HA epitopes—the Sa site (residues 160 and 163) and Ca site (residue 130), also recognized by humans. The *in vitro* neutralizing activity of the pig mAbs was comparable to the strongest human mAbs. One of the porcine mAbs, pb27, targeting the same HA1 site as 2-12C, encompassing residue K130, abolished lung and broncho-alveolar lavage (BAL) virus load and greatly reduced lung pathology after an intravenous administration at 10 mg/kg *in vivo*, although nasal shedding was not eliminated (11). Interestingly, when administered at a lower dose of 1 mg/kg, both human 2-12C and porcine pb27 significantly reduced lung pathology and lung virus load, suggesting that mAbs with powerful neutralizing activity may be effective *in vivo* at relatively low doses (10, 11).

Here we wished to determine the potency of another porcine mAb, pb18, against the Sa site encompassing residue K160, recognized by many human sera and mAbs. We tested the prophylactic administration of pb18 at 15 and 1 mg/kg. We also asked whether the effect of pb18 may be augmented additively or synergistically by the simultaneous administration of pb27, which targets the epitope encompassing K130.

## Materials and Methods

### Ethics Statement

Animal experiments were approved by the Pirbright Institute Ethics Committee and the Animal and Plant Health Agency (APHA) according to the UK animal (Scientific Procedures) Act 1986 under project license P47CE0F2. Both Institutes conform to the ARRIVE guidelines.

### Monoclonal Antibodies

The generation of porcine H1N1pdm09-specific mAbs was described previously (11). For animal studies, pb18 and pb27 were produced in bulk by Absolute Antibody Ltd (Redcar, UK) and dissolved in 25 mM Histidine, 150 mM NaCl, 0.02% Tween, pH 6.0 diluent.

### Animal Studies

Twenty 5-week old, Landrace × Hampshire cross female pigs were obtained from a commercial high-health status herd and were screened for antibody-free status against four swine influenza virus antigens: H1N1pdm09, H1N2, H3N2, and avian-like H1N1. Pigs weighed between 8 and 11 kg (average 9.5 kg). Pigs were randomized into four groups of five animals: the first group received 15 mg/kg of pb18; the second group received 1 mg/kg of pb18; the third received a combination of 1 mg/kg of pb27 and 1 mg/kg pb18 and; the fourth control group was left untreated. The mAbs were administered to the ear vein of the animals sedated with stresnil (Janssen pharmaceuticals). Twenty-four hours after mAb administration, all animals were challenged intranasally with 1 × 10^6^ PFUs of pandemic swine H1N1 isolate, A/swine/England/1353/2009 (H1N1pdm09) in 2 ml (1 ml per nostril) using a mucosal atomization device (MAD300; Wolfe Tory Medical). Clinical signs (temperature, loss of appetite, recumbence, skin hemorrhage, respiratory change, nasal discharge, and altered behavior) were observed and recorded. Clinical signs were mild and no animal developed a moderate or severe disease.

### Gross Pathology and Histopathological Scoring of Lung Lesions

At *postmortem*, the lungs were removed and digital photographs were taken of the dorsal and ventral aspects. Gross pathology was scored by quantitating the lesion areas as previously described by Halbur (12). Lung tissue samples from the right cranial, middle, and caudal lung lobes were excised and fixed in 10% neutral buffered formalin for routine histological processing. Formalin-fixed tissues were paraffin wax-embedded and 4 µm sections were cut and stained with hematoxylin and eosin (H&E). Immunohistochemical detection of influenza A virus nucleoprotein (NP) was performed as previously described (13). Histopathological changes in the H&E-stained lung tissue sections were scored by a veterinary pathologist blinded to the treatment group using five parameters (necrosis of the bronchiolar epithelium, airway inflammation, perivascular/bronchiolar cuffing, alveolar exudates, and septal inflammation) scored on a 5-point scale of 0 to 4 and then summed to give a total slide score ranging from 0 to 20 per slide and a total animal score from 0 to 60 (14). These scores together with the extent of NP staining quantified in the same way were displayed as the Iowa histopathology score (15).

### Tissue Sample Processing

Two nasal swabs (one per nostril) were taken each day following the challenge with H1N1pdm09. The swabs were placed into 2 ml of virus transport medium (VTM) comprising tissue culture medium 199 (Sigma-Aldrich) supplemented with 25 mM 4-(2-hydroxyethyl)-1-piperazineethanesulfonic acid (HEPES), 0.035% sodium bicarbonate, 0.5% bovine serum albumin, penicillin 100  IU/ml, streptomycin 100 µg/ml, and nystatin 0.25 µg/ml, vortexed, centrifuged to remove debris, aliquoted and stored at −80°C for subsequent virus titration. Serum samples were collected at the start of the study (prior to mAb administration and challenge) and at 0, 1, 3, and 4 DPI of the challenge. Broncho-alveolar lavage fluid (BAL) was collected from the entire left lung with 100 ml of 0.1% BSA+PBS. BAL samples were centrifuged at 300×*g* for 15 min, supernatant was removed, aliquoted, and frozen for antibody and virus titer analysis. The accessory lung lobes were dissected out and frozen at −80°C for subsequent virus titration. The whole lobe was cut into pieces and 10% (w/v) pieces of the lung were homogenized in 0.1% BSA using the gentle MACS Octo dissociator, the homogenate was clarified by centrifugation and the supernatant was used for virus titration.

### Virus Titration

Virus titers in nasal swabs, BAL fluid, and accessory lobe were determined by plaque assay on MDCK cells. The samples were 10-fold serially diluted in Dulbecco’s Modified Eagle’s Medium (DMEM) and 200 µl overlaid on confluent MDCK cells in 12-well tissue culture plates. After 1 h, the wells were washed and overlaid with 1 ml of 0.6% agarose in culture medium with 2 µg/ml of TPCK (Tosyl phenylalanyl chloromethyl ketone) trypsin. Plates were incubated for 48 h at 37°C. The overlay was removed, and plaques were visualized by staining the monolayer with 0.1% (v/v) crystal violet and enumerated.

### HA Gene Sequencing

Nasal swabs were collected in Trizol at 4 DPI and stored at −80°C. Subsequently, 0.4 ml aliquots were thawed and extracted with 90 µl of chloroform. Following centrifugation at 12,000 rpm/15 min, the aqueous phase (~250 µl) was transferred to a 2 ml Eppendorf tube and 1.5 vol (375 µl) 100% ethanol added, mixed by inversion, then centrifuged briefly. The liquid was transferred to a vRNA capture column (Qiagen: QIAamp viral RNA mini kit; #52906) and extracted following the manufacturer’s instructions before eluting in 50 µl of supplied AVE-buffer. RT-PCRs were performed using QIAGEN OneStep *ahead* RT-PCR kits (#220213) to amplify HA-gene products for Sanger sequencing and whole genome products for Illumina MiSeq sequencing (primer sequences available on request). Amplification products were then purified, sequenced, curated, and analyzed as described recently using the Staden package for Sanger sequencing and an in-house pipeline developed to analyze MiSeq outputs (16).

### Quantitation of mAbs in Serum Samples

Quantification of administered mAbs, pb18, and pb27, in serum, BAL and nasal swabs were determined by ELISA. Ninety-six well microtiter plates (Maxi Sorp, Nunc, sigma-Aldrich, UK) were coated with 1 µg/ml of HA in PBS overnight at 4°C. Plates were blocked with 200 µl of blocking solution composed of 4% milk powder in PBS, supplemented with 0.05% Tween-20 (PBS-T) for 2 h at room temperature. A standard curve for pb18 mAb was prepared as 1:2 serial dilutions starting at 500 ng/ml in dilution buffer and added in duplicate to the assay plate. Samples and standard were incubated for 1 h at room temperature. The plates were washed four times with PBS-T and incubated with detecting antibodies; polyclonal goat-anti pig IgG HRP (1:20,000) (Bio-Rad). Binding of Abs was detected by developing with 50 µl/well 3,3’,5,5’-tetramethylbenzidine (TMB) high sensitivity substrate solution (Biolegend, UK) and stopping with 50 µl 1 M sulfuric acid. The plates were read at 450 and 630 nm with the Cytation3 Imaging Reader (Biotek).

### Microneutralization Assay

Neutralizing antibody titers were determined in serum using a microneutralization (MN) assay. Briefly, viruses were diluted in virus growth medium (VGM; DMEM–Penicillin–streptomycin–0.1% BSA) and titrated to give plateau expression of NP in 3 × 10^4^ MDCK-SIAT1 cells after overnight infection in 96-well flat-bottomed plates. Sera were heat inactivated at 56°C for 30 min. Sera were diluted in 50 µl of VGM to 1:4 and serially double diluted. Approximately 50 µl of diluted virus was added to the serum and incubated for 1 h at 37°C. In each well 100 µl of 3 × 10^4^ MDCK-SIAT1 cells were added and incubated overnight at 37°C. The monolayer was fixed with 4% paraformaldehyde and permeabilized with Triton-X100 and stained with mouse anti-NP IgG1 (clone AA5H, Bio-Rad Antibodies) followed by goat anti-mouse HRP (DAKO) antibody. TMB substrate was added and incubated for 2–5 min and the reaction was stopped with 50 µl of 1 M sulfuric acid and absorbance was measured at 450 and 630 nm (reference wavelength) on the Cytation3 Imaging Reader (Biotek). The 50% inhibition titer was defined as the final dilution of serum that caused ≥50% reduction in NP expression.

### Statistical Analysis

**Figure 1:**
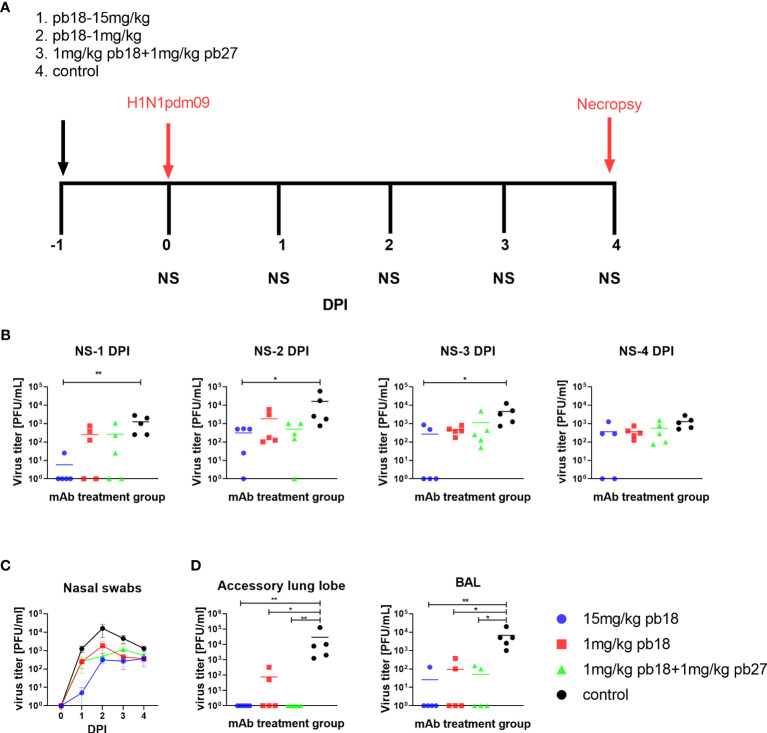
Experimental design and virus load. pb18 was administered at 15 mg/kg, 1 mg/kg or simultaneously with pb27 at 1 mg/kg each to pigs which were challenged with H1N1pdm09 virus 24 h later. The control animals were untreated. Nasal swabs (NS) were taken at 0, 1, 2, 3, and 4 DPI, and the pigs were culled at 4 DPI **(A)**. Virus load in daily nasal swabs **(B)** and over time **(C),** accessory lung and BAL at 4 DPI **(D)** were determined by plaque assay. Each data point represents an individual animal within the indicated group and the mean is shown as a horizontal line. The time course plots **(C)** show the mean (line) and standard error of mean (SEM) for each group of five pigs. Asterisks denote significant differences *p <0.05, **p <0.01, versus control as analyzed by Kruskal–Wallis test with Dunn’s multiple comparison test.

**Figure 2:**
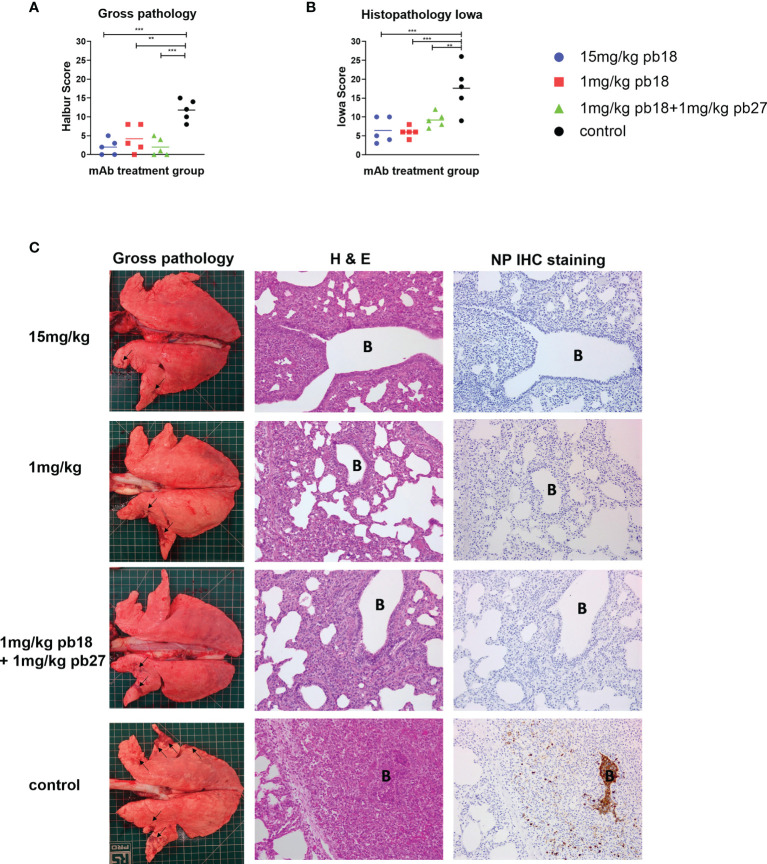
Lung pathology. pb18 was administered at 15 mg/kg, 1 mg/kg or simultaneously with pb27 at 1 mg/kg each to pigs which were challenged with H1N1pdm09 virus 24 h later. The control group animals were untreated. The animals were culled four days later and the lung scored for appearance of gross and histopathology lesions. The gross and histopathology scores for each individual in a group and the group means are shown **(A, B)** Representative gross pathology, histopathology (H&E staining; original magnification 100×), and immunohistochemical NP staining (original magnification 200×) for each group are shown **(C)** Bronchiole region is depicted as “B”. The histopathological Iowa scores include the NP staining. Pathology scores were analyzed using the one-way ANOVA with Bonferroni’s multiple comparison test. Asterisks denote significant differences **p <0.01, ***p <0.001 versus control.

**Figure 3:**
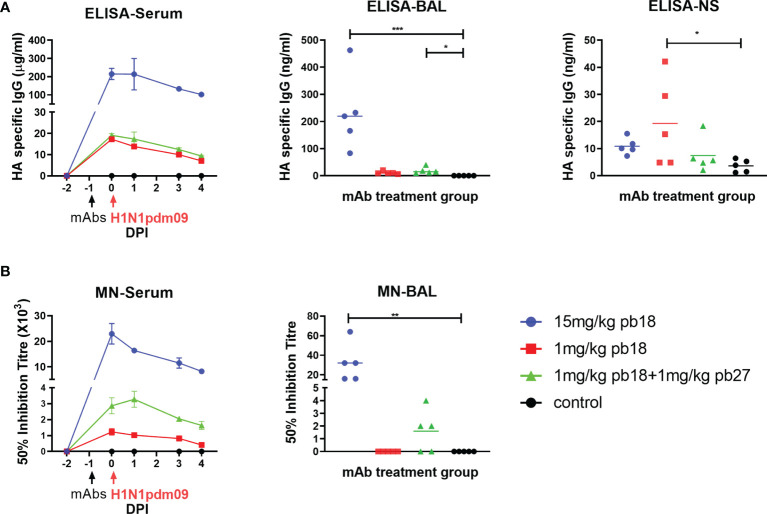
Concentration and neutralization titers of mAbs in serum, BAL and nasal swabs. HA specific IgG in sera was assessed by ELISA at the indicated DPI and in BAL and nasal swabs (NS) at 4 DPI **(A)**. The 50% neutralization titers against H1N1pdm09 in sera at the indicated timepoints and in BAL at 4 DPI are shown **(B)**. The time course plots (serum) show the mean (line) and SEM (error bars) for each group of five pigs. The dot plots (BAL and NS) show the data for individual pigs with a group and the horizontal line shows the mean for the group. Data were analyzed using a Kruskal–Wallis test with Dunn’s multiple comparison test. Asterisks denote significant differences *p <0.05, **p <0.01, ***p <0.001 versus control.

Statistical analyses were performed using Graphpad Prism 8.3 (GraphPad software). Normally-distributed data sets were subjected to a one-way ANOVA with Bonferroni’s multiple comparisons test (**Figure 2**), while non-normally distributed data sets were subjected to a Kruskal–Wallis test with Dunn’s multiple comparisons test (**Figures 1** and **3**). Significant differences were depicted on the graphs as *p <0.05, **p <0.01, and ***p <0.001.

## Results

### mAb Administration Reduced Virus Load

To determine the *in vivo* efficacy of porcine mAbs, pb18 was administered at 15 mg/kg, 1 mg/kg or in combination with pb27 at 1 mg/kg each (pb18 + pb27). Control animals were untreated. Twenty-four hours after mAb administration, all the animals were challenged with H1N1pdm09 and culled at 4 days post infection (DPI) (**Figure 1A**). The virus load in nasal swabs was assessed daily over the 4 days by plaque assay. Nasal virus loads were significantly decreased at 1, 2, and 3 DPI in the 15 mg/kg pb18 group and when assessed by the area under the curve (AUC) compared to the control group (p = 0.004) (**Figures 1B, C** **)**. The AUC for the animals treated with combined pb18 + pb27 at 1 mg/kg was also reduced (p = 0.056). However, 1 mg/kg of pb18 alone was insufficient to reduce nasal virus shedding (p = 0.126). We also assessed virus load in BAL and lung at 4 DPI (**Figure 1D**). No virus was detected in the lungs of the 15 mg/kg pb18 and pb18 + pb27 treated groups at 4 DPI, while there was virus in 2 out of 5 pigs in the 1 mg/kg pb18 group, but there were no significant differences between the treated groups. Similarly, in BAL, virus load was significantly reduced in all mAb treated groups compared to the controls. Overall, the administration of pb18 at 15 mg/kg had a significant effect on virus shedding and lung and BAL virus loads, while the lower doses or combination of the two mAbs reduced lung virus and BAL loads. There was no evidence of synergistic effect of the pb18 plus pb27 cocktail.

### mAb Administration Reduced Lung Pathology

Following the challenge with H1N1pdm09, all the control animals developed typical gross lung lesions indicative of influenza infection by day 4 DPI, as previously reported (17). All single mAb treated groups and the combined pb18 + pb27 group showed significant reductions in gross pathology compared to the control group (**Figures 2A, C**). In the control animals, characteristic histological lesions of influenza infection were observed, ranging from mild to severe necrotizing bronchiolitis, with weakening of the bronchial and bronchiolar epithelium and neutrophilic exudation in bronchiolar lumina and alveoli. Areas of bronchointerstitial pneumonia with thickening of alveolar septa and peribronchial and perivascular infiltration by lymphohistiocytic cells were present. Immunolabeling for Influenza A nucleoprotein could be observed in bronchial and bronchiolar epithelium, macrophages in inflammatory exudates, and occasional pneumocytes. Compared to the control group, all mAb treated animals showed reduced severity of the pulmonary histopathological changes and lower numbers of influenza A nucleoprotein antigen immunolabeled cells (**Figures 2A, C**). The histopathological score including the NP staining (Iowa score) showed significant reductions in all mAb treated groups compared to the control (**Figure 2B**). There were no statistically significant differences between any of the mAb treated groups.

### Quantitation and Microneutralization of mAbs in Serum, BAL and Nasal Secretions (NS)

The concentration of administered mAbs in serum at 0, 1, 3, and 4 DPI was determined by ELISA using recombinant HA glycoprotein. Peak concentrations of 215, 17, and 19 μg/ml pb18 were detected in the 15 mg/kg pb18, 1 mg/kg pb18, and pb18 + pb27 groups, respectively, at 24 h after administration. A decline in serum mAb concentrations was observed over the next 4 days to 101, 9, and 7 μg/ml respectively (**Figure 3A**). BAL samples at 4 DPI showed the presence of administered mAbs; averages of 232, 9, and 18 ng/ml for 15 mg/kg pb18, 1 mg/kg pb18 and pb18 + pb27 groups, respectively. The administered mAbs were detected in nasal swabs at 4 DPI. We also analyzed the neutralizing activity of the mAbs in serum and BAL. In serum, there was 50% inhibition neutralizing titers of 1:23,000, 1:1,200, and 1:2,900 for the 15 mg/kg pb18, 1 mg/kg pb18 and pb18 + pb27 groups respectively after 24 h of administration which gradually declined over the next four days (**Figure 3B**). Microneutralization titers closely corresponded to the level of mAb in serum. Neutralizing activity in BAL was seen in all animals of the 15 mg/kg pb18 group, in three animals administered 1 mg/kg of both pb18 and pb27, but in no animals of the 1 mg/kg pb18 group. Titers in BAL are low because lavage is carried out with 100 ml PBS/BSA diluting any Ab present in the airways.

### Sequencing of Virus

The stock of challenge virus and viruses from the nasal swabs of all 20 experimental animals were sequenced by both Sanger and whole genome methods, with a focus on the HA gene. Complete HA gene sequences were recovered for the challenge virus and from 15 of the 20 animal specimens (**Table 1**). The ranges of HA nucleotide coverage from each experimental animal varied from 10–30 to 159–2,787 with no obvious association with administration, or not, of mAb. The coverage for the challenge virus stock was 1,889–4,820. The challenge virus showed polymorphism at three positions in HA1: 154, 155, and 209. The dominant amino acid at these three positions (K154, G155, and E209) was non-polymorphic in 15, 12, and 10 of the experimental animal specimens, respectively, and remained the dominant amino acid in all but one of these specimens. Animal 3194 in the 15 mg/kg pb18 group showed dominance of E155 and K209, and A186T substitution. Two additional animals in the 15 mg/kg pb18 group, 3190 and 3192, showed polymorphism at HA1 positions 184 and 186, or HA2 V201A substitution, respectively. One animal each in the 1 mg/kg pb18, p18 + p27 and the control group yielded sequences that showed polymorphism at HA1 position 222. Overall, given that the HA epitopes were recognized by the mAbs have been mapped by the selection of escape-variants (10), but with the caveat of relatively low depths of nucleotide coverage for samples from experimental animals (presumably reflecting the quality/quantity of RNAs recovered), these results indicate that the administered mAbs did not drive selection of escape-variants during the course of these studies.

**Table 1:** Virus HA amino acid variation in viruses recovered from experimental animals.

HA1 (HA2) amino acid position	154*		155*	184	186*	209*	222*	(201)	Animal no
Challenge stock amino acid	X (K65:E35)		X (G95:E5)	T	A	X (E60:K40)	G	V	
15 mg/kg pb18 group		K	G	T97:I7	A30:T70	E			3190
		K	G			E		A	3192
		K	E		T	K			3194^#^
		K	G			E			3206
1 mg/kg pb18 group		K	G			E			3195
		K	G			E			3196
		K	G			E			3197
		K	G			E70:K30	G92:V8		3199
1 mg/kg each pb18 + pb27 group		K	G			E	G60:D40		3200
		K	G			E			3201
no mAb (control) group		K	G			E91:K9			3205
		K	G			E			3207
		K	G			E			3208
		K	G55:E45			E55:K45			3209^#^
		K	G85:E15			E87:K13	G94:D6		3191

Complete HA gene sequences were recovered from 15 of the experimental animals and the A/swine/England/1353/2009 challenge virus stock. Only positions that differ from the challenge virus are indicated. *Residues where amino acid polymorphism was seen in at least one virus. ^#^For these samples complete HA sequences were recovered by Sanger sequencing only; good quality chromatograms with single well-defined peaks and high signal to noise (baseline) ratios were obtained allowing us to distinguish minority variants in the traces at frequencies down to 5%, only those polymorphisms shown for the sample from animal 3209 were observed.

## Discussion

In this study, we have demonstrated that prophylactic intravenous administration of porcine mAb pb18 at 15 mg/kg significantly reduced lung pathology and nasal virus shedding and abolished lung virus load in pigs following the H1N1pdm09 challenge. When given at 1 mg/kg, pb18 significantly reduced lung gross and histopathology and lung and BAL virus loads, but not nasal shedding of virus. Similarly, when pb18 was given in combination with pb27 at 1 mg/kg each, lung virus load and pathology were reduced, although without an apparent additive or synergistic effect.

The present data are in agreement with our previous experience with the human 2-12C and porcine pb27 mAbs in similar challenge experiments (10, 11). Intravenous administration of high doses (15 mg/kg or 10 mg/kg) was required to reduce nasal virus shedding, although this was inconsistent and seldom complete, as in the present experiment. In contrast, the effect on lung pathology and lung virus load is consistent and is also seen at one log lower doses in all experiments. This is in accord with our previous study using DNA encoded human 2-12C, in which the serum concentration of the DNA encoded mAb was 100 times lower than in pigs given 2-12C mAb intravenously at 15 mg/kg, but we observed significant protection against disease pathology in the lungs (10). These results indicate that a lower dose is sufficient to reduce the severity of disease and lung virus load but to prevent virus shedding from the upper respiratory tract and transmission other measures are needed. The use of a different IgG subclass or local administration to the respiratory tract might be more effective in suppressing nasal virus shedding (18). It is also interesting to note that the effect of low dose mAb in reducing lung pathology and virus load but not virus shedding, is very similar to the effect of the powerful influenza specific CD8 responses induced by immunization with the single cycle influenza vaccine candidate S-FLU in pigs (19, 20). This contrasts strongly with the effect of cytotoxic T cells in mice, which have been shown to protect both against disease (weight loss) and to reduce viral load (21–23). This is an important difference between large and small animal models, and it will be important to determine which best predicts the outcome of therapy in humans.

Despite the development of many human mAbs, very few are used to treat or prevent infectious diseases though mAbs to respiratory syncytial virus, anthrax, and *Clostridioides difficile* have been shown to be effective (24). Two different monoclonal antibody products have been shown to reduce mortality from the Ebola virus (25) and more recently from COVID-19 (26). Because of the antigenic variability of influenza viruses, efforts to use mAbs for therapy have concentrated on those that recognize epitopes that are conserved between strains. MAb MHAA4549A binds to a conserved epitope on the stem HA and neutralizes all human A strains (27); VIS410 binds to both group 1 and group 2 HAs of influenza A viruses (28); CR6261 neutralizes the virus by blocking conformation rearrangements associated with membrane fusion (29); CR8020 has neutralizing activity against most group 2 viruses (30) and TCN recognizes conformational epitopes in the ectodomain of the matrix 2 protein (31–33). Some of these have shown promise in reducing clinical signs in uncomplicated influenza but further studies are needed to demonstrate their utility in preventing morbidity and mortality in severe disease (7). Most of these studies have used mAb doses of 20–60 mg/kg, but our results suggest that a lower dose may be effective in reducing lung pathology, a feature of disease that is more difficult to assess in clinical studies.

Although significant differences in lung pathology and lung viral load compared to control untreated animals were observed, there were no differences in these parameters between the mAb treated groups. In particular there was no evidence of a synergistic or additive effect of the pb18 and pb27 cocktail, although the mAbs targeted different epitopes. This is in agreement with other studies on the use of mAb cocktails alone in influenza infection although combination therapy with the viral polymerase inhibitor favipiravir and mAbs against the receptor-binding site and stem of virus HA completely stopped virus replication in nude mice, resulting in virus clearance (3, 34, 35). Administration of both anti-HA and anti-NA antibodies might also be effective and further studies to define whether the pigs generate broadly inhibiting anti-NA antibodies as has been shown in humans, would allow us to test how protective these are *in vivo* (36).

In contrast, in the Ebola virus infection, a high-resolution structural analysis revealed a mechanism of cooperativity of mAb cocktails (37). A two-antibody cocktail offered protection in mice against the most antigenically divergent virus and demonstrated high therapeutic efficacy against live EBOV challenge in non-human primates. These findings offered a rational strategy for the development of a potent two-antibody cocktail design based on structural features of mAb interactions with EBOV and the use of cocktails may result in lowered infection rates and virus loads, thereby reducing the probability of neutralization-escape variants emerging (38–41). However, in other systems molecular interactions in which two or more mAbs recognize the same antigen synergistically are poorly defined, but in the future such interactions might contribute greatly to the overall efficacy of protective mAb cocktails (42–45).

In the present study we did not use an irrelevant isotype-matched control mAb, but we have tested such a control in a previous study with the human 2-12C and the isotype matched human IgG1 did not show any effect in reducing pathology or viral load (10). However, further experiments should examine the effect of an irrelevant porcine mAb control. In our experiment, the mAbs were given 24 h before virus challenge, while in humans mAbs will usually be administered post-infection. Further experiments will be needed to determine the efficacy of mAb therapy after infection in the porcine model and the pharmacokinetic properties of the mAbs. The pig influenza model could also be used to evaluate antibody delivery platforms, the effect of IgA and different IgG subclasses and the role of Fc-mediated functions.

Three distinct polymorphisms were identified in the challenge virus in HA1 at positions 154, 155, and 209 and additional polymorphism or substitution was seen at positions 184, 186, or 222 in some of the samples from experimental animals. While there was no evidence for direct mAb-driven evolution of the virus HA gene in any of the experimental animals, amino acid substitutions at the equivalents of all six of these positions have been associated with antigenic drift/mAb escape and/or changes in receptor specificity/host range in at least one influenza A HA subtype. Notably, substitutions at positions 155 (46–49), 186 (50–52), 209 (53), and 222 (54, 55) in the HA of A(H1N1)pdm09 viruses have been associated with such effects. The apparent lack of mAb driven HA evolution may partly be explained by the fact that samples were taken at only 4 DPI and tight bottlenecks may occur with very few viruses initiating infection (56). Such a scenario could explain the HA sequence derived from animal 3194 with HA1 G155E, A186T, and E209K amino acid substitutions, the infecting virus being a minor variant within the A/swine/England/1353/2009 challenge virus stock.

In summary, we have shown that the pig is a useful model to test mAb delivery and efficacy, particularly for influenza virus, since pigs are infected with the same H1N1pdm09 influenza A viruses as humans. We have shown that pigs mount very similar antibody responses to the virus’ HA as humans (11). The lobar and bronchial structure of the pig lung is very like that of humans and here we showed that a low dose of mAb may be highly effective in preventing lung pathology and severe disease. Future clinical studies should take into account the possibility that mAb therapy may be highly effective in preventing severe disease without affecting virus shedding.

## Data Availability Statement

The original contributions presented in the study are included in the article/supplementary material. Further inquiries can be directed to the corresponding authors.

## Ethics Statement

The animal study was reviewed and approved by the Pirbright Institute Animal and Plant Health Agency (APHA).

## Author Contributions

ET, BP, AT, and JH conceived, designed and coordinated the study. BP, AM, and BC, performed animal experiments, processed samples and analyzed the data. AN carried out postmortem and pathological analyses. AT and PR designed experiments, provided reagents and developed assays for assessment of antibody function. RD and JM performed sequencing analysis. ET, BP, PR and RD wrote and revised the manuscript. All authors contributed to the article and approved the submitted version.

## Funding

This work was supported by the Bill & Melinda Gates Foundation grants OPP1201470 and OPP1215550 (Pirbright Livestock Antibody Hub); the UKRI Biotechnology and Biological Sciences Research Council (BBSRC) grants BBS/E/I/00007031, BBS/E/I/00007038, and BBS/E/I/00007039. AT and PR were funded by the Chinese Academy of Medical Sciences (CAMS) Innovation Fund for Medical Sciences (CIFMS), China Grant 2018-I2M-2-002, the Townsend-Jeantet Prize Charitable Trust (charity number 1011770) and the Medical Research Council (MRC) Grant MR/P021336/1. The Worldwide Influenza Centre is supported by the Francis Crick Institute receiving core funding from the Cancer Research UK (FC001030), the Medical Research Council (FC001030) and the Wellcome Trust (FC001030).

## Conflict of Interest

The authors declare that the research was conducted in the absence of any commercial or financial relationships that could be construed as a potential conflict of interest.

## Publisher’s Note

All claims expressed in this article are solely those of the authors and do not necessarily represent those of their affiliated organizations, or those of the publisher, the editors and the reviewers. Any product that may be evaluated in this article, or claim that may be made by its manufacturer, is not guaranteed or endorsed by the publisher.
